# BUILDING AN AGE-FRIENDLY HEALTH CARE SYSTEM

**DOI:** 10.1093/geroni/igz038.666

**Published:** 2019-11-08

**Authors:** Janice Lundy

**Affiliations:** Perry County Memorial Hospital, Perryville, Misouri, United States

## Abstract

Gateway GWEP has partnered with a rural Missouri critical access hospital to establish an age-friendly health system in their community. Program innovations include development and training of: 1) electronic health records integration of the Rapid Geriatric Assessment (RGA) for all patients 65+ years old; 2) RGA-based protocol for Medicare Annual Wellness Visits (MAWV); 3) Multidisciplinary health care assessment team 4) Evidence-based or Evidence Informed treatment interventions, including Cognitive Stimulation Therapy (CST), exercise and strengthening program for persons participating in CST, Care of Persons with Dementia in their Environments (COPE) and caregiver support. Since 2015, 1,200 RGAs, 338 MAWVs have been completed; 165 persons with dementia participated in CST; and 74 have participated in exercise and strengthening. Data supports positive outcomes in functional independence, cognitive status, and care-giver dementia management skills and well-being. Successes and lessons learned regarding strategies to develop an age-friendly health systems will be discussed.

